# The Natural History of Obstructive Sleep Apnea: A Scoping Review

**DOI:** 10.3390/healthcare14030325

**Published:** 2026-01-27

**Authors:** Alexandros Kalkanis, Theodoros Panou, Kostas Archontogeorgis, Paschalis Steiropoulos

**Affiliations:** 1 Department of Respiratory Diseases, Leuven University Center for Sleep and Wake Disorders, University Hospitals Leuven, UZ Leuven, Herestraat 49, 3000 Leuven, Belgium; 2 Department of Pneumonology, Medical School, Democritus University of Thrace, 68100 Alexandroupolis, Greece; theopano2@med.duth.gr (T.P.); steiropoulos@yahoo.com (P.S.); 3 MSc “Sleep Medicine”, Medical School, Democritus University of Thrace, 68100 Alexandroupolis, Greece; k.archontogeorgis@yahoo.it

**Keywords:** obstructive sleep apnea, natural history, disease progression

## Abstract

**Highlights:**

**What are the main findings?**

- The natural history of OSA reveals that while many children experience spontaneous remission, a substantial proportion develop persistent or recurrent disease that continues into adolescence and adulthood.
- In adults, OSA is predominantly a chronic and progressive condition influenced by anatomical factors, obesity, aging, and hormonal changes.

**What are the implications of the main findings?**

- Monitor children with OSA through adolescence to detect recurrence early.
- Reduce adult OSA risk with weight loss, CPAP adherence, and comorbidity control.
- Manage OSA as a lifelong condition requiring prevention and regular follow-up.

**Abstract:**

Obstructive sleep apnea (OSA) is a common disorder caused by recurrent upper airway obstruction during sleep, affecting individuals across the lifespan. In children, OSA commonly results from adenotonsillar hypertrophy and may resolve spontaneously or following surgical intervention. Among adolescents and adults, OSA is more frequently associated with modifiable lifestyle factors, particularly obesity. The natural history of OSA may evolve from intermittent snoring and mild disease to moderate or severe forms if left untreated, leading to reduced health-related quality of life and overall health deterioration. Early identification of OSA, especially in mild and moderate cases, allows timely interventions to improve OSA-associated indices and may prevent progression to severe disease. Continuous positive airway pressure therapy remains the treatment of choice for adults, providing effective symptom control and reducing long-term complications, although adherence rates vary. In obese patients, sustained weight reduction represents the most effective disease-modifying strategy: a ≥5% weight loss is associated with an approximately 80% reduction in progression risk, while bariatric surgery achieves remission in up to 60–65% of cases at one year. Emerging anti-obesity pharmacotherapies have also demonstrated clinically meaningful reductions in the apnea–hypopnea index. Comorbid conditions such as hypertension, type 2 diabetes, and depression exacerbate OSA severity, impair treatment response, and complicate overall disease management. This review uniquely integrates pediatric and adult longitudinal data, treatment-modified trajectories, and emerging therapeutic approaches to provide a life-course perspective on OSA natural history, highlighting opportunities for early, phenotype-directed intervention to possibly alter disease course and long-term outcomes.

## 1. Introduction

Obstructive sleep apnea (OSA) is a prevalent sleep disorder characterized by recurrent upper airway collapse during sleep, resulting in intermittent oxygen desaturation and sleep fragmentation. Although the precise mechanisms underlying upper airway collapse remain incompletely understood, OSA is thought to arise from the interplay of multiple factors, including obesity, craniofacial abnormalities, altered upper airway muscle function, pharyngeal neuropathy, and rostral fluid shifts toward the neck [1]. Traditionally regarded as a predominantly mechanical disorder, OSA is now increasingly recognized as a complex condition with important biological underpinnings. Emerging evidence implicates genetic influences, neurotransmission pathways, and the gut microbiome as contributors to its pathophysiology. Dopamine and related genetic modulators have been implicated in OSA. Elevated serum dopamine levels have been reported in patients with OSA, with concentrations independently correlating with both disease presence and male sex [2]. Genotypic analyses further identified specific allelic variations in dopamine-related genes as potential independent predictors of OSA severity [2]. Comparable findings have been reported for serotonergic signaling, with polymorphisms in serotonin receptor-encoding genes proposed to underlie the association between sleep bruxism and OSA [3]. Moreover, several key physiological processes relevant to OSA—such as muscle tone, genioglossus activity, and respiratory control—are modulated by serotonergic pathways [4]. In parallel, growing interest has focused on the role of the gut microbiome, which comprises bacteria, viruses, and fungi that colonize the gastrointestinal tract and influence immune regulation, metabolism, nutrient absorption, and organ development. In animal models, chronic intermittent hypoxia and chronic sleep fragmentation—two hallmark pathological features of OSA—have been shown to adversely alter fecal microbiota composition and function, as well as host metabolic profiles [5]. Additionally, specific bacterial families and microbially derived bile acids appear to modulate OSA-induced atherosclerosis, a highly prevalent comorbidity in these patients [6].

It represents a major global health burden, affecting over one billion individuals worldwide, primarily middle-aged and older adults [7,8]. Beyond its high prevalence, OSA significantly impairs health-related quality of life (HRQoL), particularly as it progresses to severe stages. OSA is not only a sleeping disorder; it is a systemic condition that contributes to cardiovascular [9], metabolic [10], and neurocognitive dysfunction [11,12,13].

Although OSA is a well-recognized disorder with significant clinical consequences, there remains a notable gap in the literature on its natural history. Specifically, high-quality longitudinal studies that follow individuals with untreated OSA over extended periods are limited. Most existing research is cross-sectional or short-term, focusing on associations between OSA and adverse outcomes rather than documenting how the condition evolves in individuals over time. As a result, key aspects such as the trajectory of disease progression, modifying factors, temporal relationships, subclinical stages, and outcome prediction remain poorly defined. Understanding the natural history of OSA—from onset and early pathogenesis to clinical manifestations, progression, and long-term outcomes—is central to developing effective preventive and therapeutic strategies. The natural history framework allows clinicians to classify disease stages and phenotypes, each associated with distinct risks and consequences [7].

Limited progress in understanding OSA’s pathophysiology and natural course, like mechanisms of disease initiation, longitudinal neurocognitive outcomes, and untreated long-term natural history, may partly be attributed to the widespread adoption of positive airway pressure (PAP) therapy. Since its introduction in 1981, PAP has dramatically reduced OSA-related morbidity and mortality [14,15]. However, its effectiveness has sometimes diverted attention from prevention and early-stage detection [7].

A comprehensive understanding of the natural history of OSA, including its onset, progression, potential remission, and long-term sequelae, remains a key research priority. Deeper insight into these aspects is essential for designing cost-effective, targeted, accessible, and easily implementable interventions that can alter disease course and improve long-term outcomes [16].

This review was conducted as a scoping review to map and synthesize the literature describing the natural history of OSA across the lifespan, and to organize evidence on trajectory patterns (onset, persistence, remission, progression, recurrence) and their determinants and modifiers (phenotypes, risk factors, comorbidities, and treatment-era considerations).

## 2. Materials and Methods

The methodological approach was informed by established scoping-review guidance and is reported in accordance with PRISMA-ScR (Preferred Reporting Items for Systematic Reviews and Meta-Analyses extension for Scoping Reviews). Because the topic spans multiple age groups, diagnostic eras, and clinical contexts, a scoping approach was selected to (i) identify and map the breadth of evidence, (ii) distinguish studies providing longitudinal trajectory data from those offering contextual or mechanistic contributions, and (iii) highlight evidence clusters and knowledge gaps without restricting inclusion to a single study design.

### 2.1. Information Sources and Search Strategy

A comprehensive literature search was conducted in Scopus, PubMed/MEDLINE, and Google Scholar from database inception through September 2025 [17]. Search strategies combined controlled vocabulary terms (including MeSH where applicable) with free-text keywords related to OSA and disease course. Core concepts included “obstructive sleep apnea” OR “OSA” combined with terms capturing longitudinal course (e.g., “natural history,” “longitudinal,” “cohort,” “follow-up,” “incidence,” “remission,” “progression”). Database-specific strategies were adapted to each platform’s indexing structure and syntax and are provided in the Supplementary Materials.

Google Scholar was included as a supplementary capture tool, not as a stand-alone indexing database. Its role was to improve sensitivity for (i) ahead-of-print/early online items, (ii) relevant cohort reports that are variably indexed across bibliographic databases, and (iii) citation-chaining (forward discovery of follow-up publications from landmark cohorts). To reduce noise and maintain interpretability, Google Scholar screening was limited to the most relevant result pages using the same concept structure as the indexed-database searches, and eligibility was restricted to peer-reviewed English-language publications.

Given the breadth of the topic and the original narrative scope of the work, no date limits or study-design limits were applied at the search stage. Reference lists of key articles and closely related reviews were manually screened to identify additional relevant sources.

### 2.2. Eligibility Criteria

Eligible records were peer-reviewed, English-language studies that contributed evidence relevant to the life-course evolution of OSA. Both pediatric and adult populations were eligible. To support the scoping objective, studies were eligible if they informed at least one of the following mapped domains:

1. Trajectory evidence: onset, persistence, remission, progression, recurrence, or long-term sequelae linked to earlier OSA;
2. Determinants/modifiers of course: phenotypes (e.g., REM-related patterns), risk factors (e.g., adiposity measures, tonsillar factors), demographic modifiers (e.g., sex/menopause), comorbidity interactions, and treatment-era considerations that plausibly shape observed trajectories or long-term outcomes.

Conference abstracts, editorials, and narrative opinion pieces were excluded. Narrative and systematic reviews were not included as primary evidence, but were used where relevant to support citation-chaining and background framing.

### 2.3. Study Selection

After duplicate removal, titles/abstracts were screened for relevance, followed by full-text review of potentially eligible records. Screening was performed independently by two reviewers, with disagreements resolved through discussion and consensus; a third reviewer adjudicated unresolved discrepancies.

**Figure 1:**
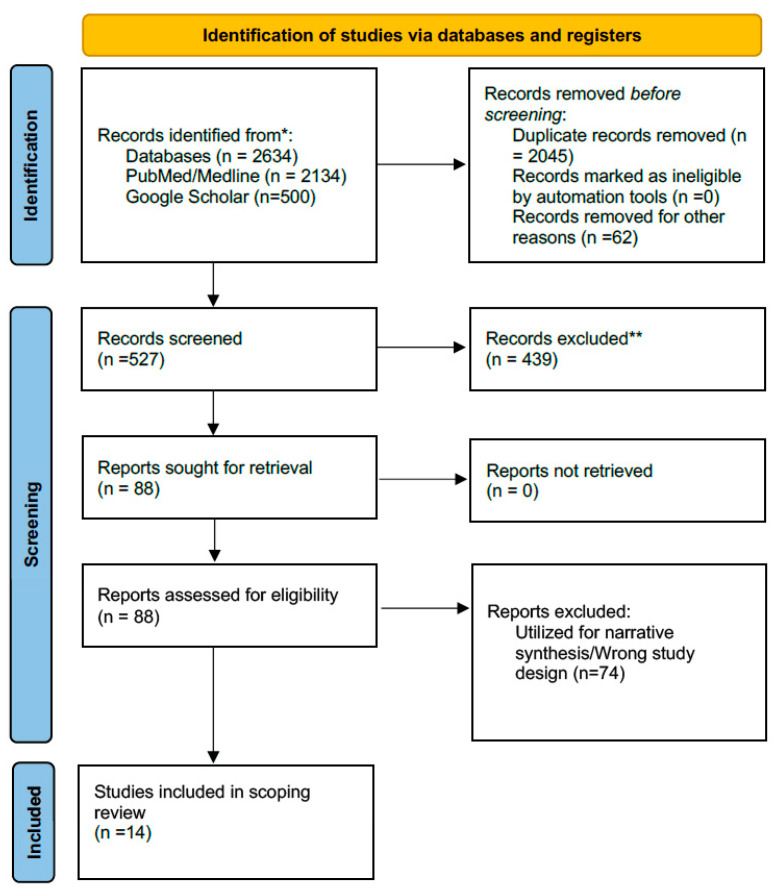
PRISMA 2020 flow diagram. * Databases searched included PubMed/MEDLINE and Google Scholar. ** Records were excluded based on predefined eligibility criteria.

The study-selection process is summarized in the PRISMA-ScR flow diagram (Figure 1). To improve transparency, exclusions at the full-text stage were grouped into prespecified categories (e.g., not OSA, not relevant to disease course/modifiers, non-peer-reviewed, non-English, and no usable course-related outcomes).

### 2.4. Evidence Organization, Data Charting, and Synthesis

A priori data-charting fields were defined to extract study-level characteristics relevant to longitudinal course mapping: setting, population, OSA definition, baseline severity metrics, follow-up duration, trajectory-related outcomes (remission/persistence/incidence/progression/recurrence), and reported predictors/modifiers. Nevertheless, this review remained narrative in nature and does not aim to perform a quantitative synthesis.

## 3. Results

### 3.1. Search Results and Study Characteristics

The database search identified 2634 records. After removing 2045 duplicates, 527 records were screened by title and abstract. Full texts were assessed for eligibility (*n* = 88), of which 74 were excluded. Fourteen studies met the criteria for inclusion in the core longitudinal trajectory evidence set and were included in the scoping review charting table (Figure 1).

Given the heterogeneity of study designs addressing OSA across the lifespan, the results are structured to reflect two complementary layers of evidence. First, a core longitudinal layer comprises studies with extractable follow-up data that directly describe OSA trajectories over time, including remission, persistence, incidence, progression, recurrence, or within-person change in severity or phenotype. These studies form the basis of the structured scoping synthesis and are summarized in Table 1. Second, a mapped contextual layer comprises additional eligible studies that inform the interpretation of trajectory patterns by elucidating developmental phenotypes, biological and behavioral modifiers, comorbidity interactions, and treatment-era influences across life stages. While these studies do not uniformly provide chartable longitudinal endpoints, they contribute essential contextual evidence and are synthesized narratively within the age- and factor-specific subsections that follow.

Across the charted longitudinal evidence base, trajectory data were available for (i) pediatric and community-based cohorts, describing remission, persistence, incidence, and progression from early childhood through adolescence and young adulthood, often alongside modifiers such as sex, adiposity, and tonsillar factors; and (ii) adult cohorts and clinical follow-up studies, evaluating longer-term stability or progression of disease severity indices, symptom trajectories, and cardiometabolic sequelae. Additional studies linked early-life OSA exposure to later blood-pressure outcomes and documented recurrence following adenotonsillectomy where relevant. Collectively, these studies represent the core empirical evidence underpinning the mapped natural history of OSA across the lifespan (Table 1).

**Figure 2:**
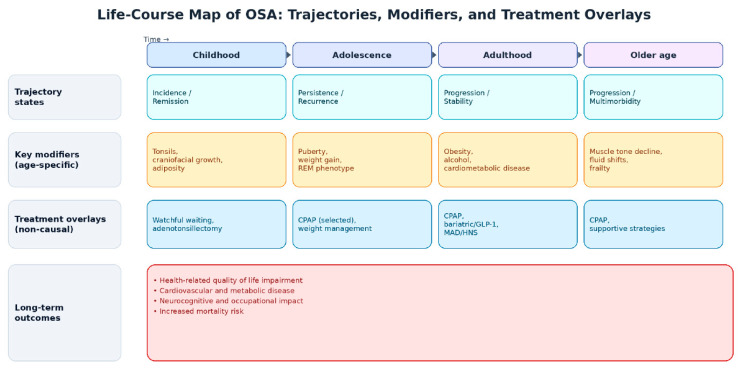
Life-course map of OSA trajectories, modifiers, and treatment overlays, as synthesized from the broader mapped literature. The figure is intended as an expert-opinion evidence map rather than a causal model.

It is worth noting here that studies on the natural disease course of OSA into adulthood are rare due to significant methodological and ethical constraints. A fundamental understanding of disease progression ideally requires longitudinal observation of untreated individuals from disease inception. However, the established morbidity of OSA, including its associations with cardiovascular disease and neurocognitive outcomes [9,11], ethically mandates treatment upon diagnosis, thereby inhibiting the observable natural course. Furthermore, the typical study of the condition only after clinical presentation (due to symptomatology) creates an evidence asymmetry across age groups. The pediatric literature, while also limited, more frequently captures incident cases due to targeted screening of high-risk populations (e.g., children with adenotonsillar hypertrophy or craniofacial syndromes) and offers some insight into early disease evolution. In contrast, adult studies almost exclusively examine prevalent disease, obscuring a coherent picture of OSA progression across the lifespan. Figure 2 visually maps age-dependent OSA trajectories alongside key modifiers and treatment-era overlays across the lifespan. The figure integrates evidence from the charted longitudinal studies (Table 1) with the broader mapped contextual literature synthesized in the sections below.

### 3.2. OSA Natural History in Children or Adolescents

The third edition of the International Classification of Sleep Disorders redefined pediatric OSA, replacing older terms such as upper airway resistance syndrome and Pickwickian syndrome. According to these updated criteria, a diagnosis of pediatric OSA requires the presence of daytime and/or nighttime symptoms along with objective polysomnographic findings. Under this revised definition, pediatric OSA is most frequently diagnosed in children aged 2–8 years, though it can occur from infancy through adolescence [18]. This developmental period is characterized by substantial physiological changes, resulting in a heterogeneous spectrum of pediatric OSA phenotypes [18]. The disorder arises from multifactorial pathophysiological mechanisms, including tonsillar hypertrophy, altered neuromuscular tone, and increased airway resistance [19].

Early observational studies based on parent-reported symptoms documented a decline in daytime sleep as children aged, despite the presence of snoring, which is often considered a hallmark of deep sleep [20]. The Cleveland Children’s Sleep and Health Study, a population-based cohort of 490 children aged 8–11, reported a baseline OSA prevalence of 4.7%, which slightly decreased to 4.3% at follow-up. Of the 23 children initially diagnosed with OSA, only 2 (8.6%) retained the diagnosis at follow-up, whereas 19 new cases emerged during the study period [21]. Subsequent analyses identified several predictors for OSA development in adolescence, including male sex, higher BMI Z-scores, and a history of tonsillectomy or adenoidectomy. Additional risk factors included upbringing in socioeconomically disadvantaged neighborhoods, African American ethnicity, and premature birth [21].

Further research reinforced the association between pediatric sleep-disordered breathing (SDB) and rapid weight gain, obesity, and African American ancestry. These factors may predispose to SDB recurrence and also diminish the long-term benefits of adenotonsillectomy [22]. Primary snoring, in contrast, appears to have limited significance in disease evolution. An eight-year follow-up study found that among children with primary snoring, 30.3% continued to snore, 31.5% experienced remission, 25.8% developed mild OSA (apnea–hypopnea index, [AHI] < 5 events/hour), and 12.4% progressed to moderate-to-severe OSA (AHI ≥ 5 events/hour) [23]. Consistently, childhood obesity emerged as a major risk factor for habitual snoring and pediatric OSA [24].

Only a limited number of longitudinal studies have examined the natural history of OSA in children and adolescents [23,25]. These studies, with follow-up durations ranging from 2 to 10 years, typically involved children aged 6–13 years [25,26]. A common limitation across these studies was reduced participant retention, often due to socioeconomic constraints [23,25,27]. Nonetheless, most reported a favorable disease course, with remission occurring in 69% to 100% of children at follow-up [23,28].

One longitudinal cohort study tracked prepubertal children into their adolescence, stratifying them by baseline AHI. All of those with baseline AHI ≥ 5 events/h achieved remission during adolescence: half improved to AHI < 2 events/h, while the remainder had AHI between 2 and <5 events/h. For children with a baseline AHI of ≥2 events/h, remission occurred in 52.9%. Conversely, 36.5% of children with an initial AHI of <2 events/hour developed AHI ≥ 2 events/h during adolescence, indicating new-onset SDB in this subgroup [23]. A 10-year longitudinal study by Chan et al. examined rapid eye movement (REM)-predominant OSA (REM-OSA) in children and found persistence in 59% of cases into young adulthood, with 72% maintaining the REM-predominant pattern. REM-OSA was further linked to adverse blood pressure outcomes for these patients [27].

OSA symptoms often worsen during adolescence. In one study, 29% of the participants showed disease progression, defined as an increase in obstructive AHI (OAHI) beyond the margin of measurement error [26]. Notably, in this study, adolescence was defined as the period between ages 9 and 15 years. In the cohort of Bixler et al., 36.5% of the participants experienced an increase in AHI to >2 events/hour (from <2 in childhood), and 10.6% developed more severe OSA, defined as AHI > 5 events/hour [23]. Another longitudinal study found that 22% of the participants had OAHI > 5 events/hour at follow-up [27]. These findings suggest that pediatric and adolescent OSA are distinct clinical entities requiring separate evaluation and management [27] (Table 1).

Therefore, REM-OSA has been identified as a clinically important pediatric subtype. Chan et al. demonstrated that REM-OSA was associated with elevated nocturnal arterial blood pressure and reduced nocturnal dipping, both being predictors of future cardiovascular risk. Although conventional indices such as OAHI can effectively distinguish children with and without OSA, more nuanced assessments may be required for specific subtype differentiation, with REM-OAHI emerging as a significant disease marker [27]. Moreover, pediatric OSA has been associated with an almost threefold increase in the risk of hypertension during youth [29]. Since REM-OSA predominates in children, early recognition and treatment may mitigate not only OSA severity but also the risk of developing hypertension during adulthood [29].

Sex influences on pediatric OSA are less pronounced than in adults. Horne et al. found no significant sex differences in severity or SDB-related consequences; however, females with moderate-to-severe OSA exhibited more internalizing behavioral symptoms and higher diastolic blood pressure [30]. Conversely, another study identified female sex as a predictor of OSA remission, whereas male gender and increased BMI were associated with disease persistence [27]. As children transition into adolescence, sex-related differences become more pronounced. Adolescent males, especially those who are obese or have tonsillar hypertrophy, are more likely to develop severe OSA [31]. Li et al. further confirmed male sex as a significant predictor of OSA, alongside other variables such as age, changes in waist circumference, and tonsil size [26]. Body composition has also been extensively evaluated. While no correlation was observed between neck circumference and SDB severity, increased waist circumference, a higher ratio of android to total body fat mass, and elevated visceral adipose tissue were significantly associated with AHI ≥ 5 events/h [23,26].

The management of pediatric OSA remains somewhat ambiguous, largely due to the high rates of spontaneous remission from childhood into adulthood [23,25]. Some experts advocate for a “watchful waiting and supportive care (WWSC)” strategy, reserving interventions for children with significant symptoms or impaired HRQoL [32]. This approach is supported by findings from the Childhood Adenotonsillectomy Trial (CHAT), which assessed cognitive and behavioral outcomes, changes in BMI, AHI, and arousal index during REM sleep [32,33,34,35]. CHAT concluded that surgical treatment was not superior to WWSC in improving cognitive outcomes in school-aged children [34]. While WWSC proponents acknowledge the benefits of surgery in selected phenotypes of children with OSA, they emphasize the need to weigh these against potential complications [36]. Despite these concerns, adenotonsillectomy has been consistently associated with notable improvements in sleep quality and overall health, with a relatively low complication rate [37]. Accordingly, another expert group recommends adenotonsillectomy as the first-line treatment for pediatric OSA, aligning with guidelines issued by the American Academy of Pediatrics, the American Academy of Sleep Medicine, and the American Academy of Otolaryngology-Head and Neck Surgery [37]. Both management strategies present valid arguments, emphasizing the need for further research to guide individualized, evidence-based treatment.

A recent randomized controlled trial, the Pediatric Adenotonsillectomy Trial for Snoring, showed that adenotonsillectomy provided particular benefit for children with moderate SDB, defined as snoring on more than three nights per week for 3 months, an obstructive apnea index < 1 event/hour, OAHI < 3 events/hour, and oxygen desaturation > 90% on polysomnography. Among 459 children, early adenotonsillectomy reduced healthcare visits by 32% and prescription use by 48%, although it had a limited impact on hospitalizations and emergency department visits [38]. These findings suggest that early adenotonsillectomy offers modest but meaningful benefits for selected children with SDB compared to WWSC [38]. The impact of adenotonsillectomy on central apnea has also been explored. A meta-analysis of 1287 children reported improvements in the central apnea index following adenotonsillectomy, though the central AHI remained unchanged. This benefit was not observed in children with certain comorbid conditions [39].

Persistent OSA, previously termed unresolved OSA, may occur after tonsillectomy, especially in children with obesity, comorbidities, or craniofacial abnormalities, though otherwise healthy children can be affected as well [40]. In such cases, CPAP is generally recommended following a comprehensive polysomnographic evaluation [41], even though adherence remains a significant challenge due to mask discomfort, side effects, or pressure intolerance [40,42]. Pharmacological therapy, combining nasal mometasone and montelukast, has shown promise in improving AHI and oxygen saturation [43]. Other adjunctive interventions include positional therapy, high-flow nasal cannula therapy, orthodontic procedures, and surgical approaches, such as tongue-base reduction, uvulopalatopharyngoplasty, lingual tonsillectomy, supraglottoplasty, tracheostomy, and hypoglossal nerve stimulation [44]. These alternatives underscore the importance of tailored management and early recognition to optimize treatment outcomes.

In children and adolescents, OSA has a profound effect on HRQoL. Early studies, such as one in 2006, reported a marked impairment in HRQoL of young OSA patients (mean OSA-18 score: 82.83), which improved significantly post-surgery (mean OSA-18 score: 34.3) [45]. In a more recent study, a significant average reduction of 15.14 in mean OSA-18 score was observed following adenoidectomy or tonsillectomy [46]. Established risk factors for pediatric OSA include tonsillar and adenoidal hypertrophy, recurrent respiratory infections, obesity, and a family history of OSA, whereas white ethnicity appears to confer a protective effect [47].

Although pediatric and adult OSA are typically regarded as distinct clinical conditions, growing evidence suggests a developmental continuum. A 20-year follow-up study by Nosetti et al. found that adults with a history of severe childhood OSA had higher rates of snoring, elevated BMI, and poorer academic performance [48]. Childhood AHI also trended toward predicting adverse cardiovascular outcomes and higher Berlin questionnaire scores in adulthood [48]. Similarly, a 10-year prospective study reported that moderate-to-severe childhood OSA independently predicted hypertension in young adults (relative risk of 2.5; 95% CI: 1.2–5.3) [49]. These longitudinal findings highlight that early diagnosis and management of pediatric OSA can significantly modify long-term outcomes, improve HRQoL, and reduce future cardiovascular risk.

Genetic and familial factors are strongly associated with OSA. A tertiary care study involving 115 children and their first-degree relatives who had undergone polysomnography revealed that while only 0.9% to 4.7% of relatives had a formal OSA diagnosis, 43% exhibited at least one symptom suggestive of the condition [50]. Another study, including 157 OSA patients and 844 first-degree relatives, emphasized the role of familial craniofacial anatomical features as a risk factor for OSA development [51] (Table 2).

### 3.3. Natural History of OSA in Adults (Table 3)

#### 3.3.1. Cross-Sectional Epidemiological and Phenotypic Description of Adult OSA

OSA in adults is highly prevalent, affecting approximately 17% of women and 34% of men in the USA, with similar rates reported globally [52]. From a demographic perspective, adult OSA has no age-specific limitations, but its incidence increases with advancing age, reaching a plateau at around 65 years of age [53]. Anatomical features play a central role in OSA pathophysiology. Common structural findings include one or more upper airway obstruction sites (most commonly in the velopharynx) and dysfunction of airway dilator muscles, such as the genioglossus [54]. Dynamic anatomical changes, namely reduced pharyngeal caliber at end-expiration due to lower body pressure or extracellular fluid redistribution in the supine position, further contribute to upper airway collapse [54]. Additional structural risk factors include tonsillar hypertrophy, macroglossia, and craniofacial abnormalities such as micro- and retrognathia [55].

The most effective strategy for early OSA detection involves targeted screening of individuals presenting with suggestive symptoms or comorbidities. This initial screening can be effectively performed using symptom checklists and validated questionnaires. Typical symptoms include sleep fragmentation, excessive daytime sleepiness, fatigue, insomnia, abrupt awakenings, and loud snoring [56]. Widely used screening tools include the Berlin questionnaire, STOP and STOP-BANG questionnaires, and the Epworth Sleepiness Scale (ESS), with the STOP-BANG exhibiting the highest predictive accuracy, especially when combined with the ESS [57,58].

#### 3.3.2. Sex and Its Association with OSA in Adults

Sex and gender differences in OSA are well-documented. While male sex is a recognized risk factor for OSA, the disparity between men and women narrows significantly after menopause, likely due to hormonal changes [59]. Women with OSA present higher odds of developing comorbid metabolic syndrome, emphasizing the importance of early detection, particularly during the menopausal transition, to prevent worsening HRQoL and systemic disease progression [60].

In men, hormonal factors also play a role in the development of OSA. Male hypogonadism is both a risk factor for and a potential consequence of OSA [61]. Low testosterone levels are associated with increased obesity, which promotes upper airway obstruction and contributes to OSA pathogenesis. Conversely, OSA can suppress gonadotropin-releasing hormone and luteinizing hormone secretion, impairing Leydig cell function and further reducing testosterone levels [61]. As a result, testosterone replacement therapy is contraindicated in men with hypogonadism and concurrent OSA, as it may exacerbate the condition [61].

Menopause represents a critical transition in the onset and progression of OSA in women. Although OSA has historically been more prevalent in men, its incidence rises significantly in women after menopause, reaching comparable levels of severity and frequency [62]. This increase is attributed to the decline in estrogen and progesterone, hormones essential for maintaining upper airway muscle tone and ventilatory stability. The loss of these protective effects, combined with menopause-related increases in visceral fat, markedly elevates OSA risk regardless of overall BMI [59,62].

Postmenopausal women often present with OSA symptoms that differ from the classic manifestations. Rather than loud snoring or witnessed apneas, they are more likely to report insomnia, fatigue, and mood disturbances, symptoms that contribute to underdiagnosis and treatment delays [63]. Despite this, more than half of postmenopausal women may meet diagnostic criteria for OSA [64]. Hormonal decline and increased visceral adiposity appear to impair ventilatory control and upper airway patency [62]. If unrecognized or untreated, OSA in postmenopausal women can progress to severe disease, worsening cardiovascular and metabolic outcomes, and diminishing HRQoL. Early screening and intervention in this population are therefore crucial to improve long-term outcomes [59].

#### 3.3.3. Natural History of Untreated Adult OSA

The natural history of untreated OSA involves progression in severity and impact over time. Studies investigating this natural history in adults vary considerably in sample size, ranging from 40 to nearly 5000 participants [15,65]. Most studies have included between 40 and 100 subjects [66,67]. Participant ages in these studies spanned from 19 to 80 years, with a mean age between 47 and 55.8 years in most cohorts [66,68]. Some investigations focused on specific populations, such as veterans, limiting generalizability [68,69]. Follow-up durations also differed substantially, although a 5-year follow-up period was most commonly reported [65,67,68].

Despite this heterogeneity, a recent meta-analysis concluded that HRQoL scores among individuals with OSA ranged from 63.97 to 70.32 on a 0–100 scale, indicating moderate-to-severe impairment [70]. Middle-aged men with undiagnosed OSA experience substantial reductions in QoL, particularly in physical functioning, general health, and vitality [71]. Moreover, OSA significantly impacts employment and work performance. Excessive daytime sleepiness contributes to work disability and loss of productivity [72,73]. In one study, nearly 27% of participants reported absenteeism attributed to OSA, disproportionately affecting younger employees [74]. Individuals with OSA are also at greater risk of involuntary job loss, occupational injuries, and motor vehicle accidents [75,76]. Collectively, these findings illustrate that untreated OSA impairs functional capacity and productivity, whereas early recognition and treatment can markedly improve clinical and occupational outcomes (Table 3).

The influence of comorbidities on the natural history of OSA has been extensively investigated [65,66,68,69], and OSA is further associated with a 2- to 3-fold increase in the risk of cardiovascular and metabolic diseases [76]. Common comorbidities include coronary artery disease, stroke, hypertension, chronic obstructive pulmonary disease (COPD), asthma, diabetes, gout, various forms of arthritis, mental health disorders, and even certain types of cancer [77,78,79]. Specifically, among individuals with conditions such as hypertension, heart failure, coronary artery disease, pulmonary hypertension, atrial fibrillation, and stroke, the prevalence of OSA ranges from 40 to 80% [11]. Arterial hypertension, in particular, is the most frequently studied comorbidity, with reported prevalence rates ranging between 46% and 53.8% in longitudinal studies [65,68]. While some studies have explored other cardiovascular conditions, such as ischemic heart disease, arrhythmias, angina, and stroke, others have focused primarily on excess body weight or obesity [65,66,68,69]. However, findings across studies are limited by inadequate adjustment for confounding factors such as age and baseline OSA severity. For example, in a small-scale study by Fisher et al. [65], participants who developed hypertension or ischemic heart disease were significantly older (57 vs. 45 years, *p* < 0.05) and exhibited higher RDI values (46 vs. 23 events/h, *p* < 0.05).

Early identification and treatment are critical, as OSA not only exacerbates these comorbid conditions but also progresses more rapidly in their presence, creating a cycle of mutual reinforcement [80]. In men, untreated OSA is strongly associated with increased cardiovascular mortality [81]. Timely diagnosis of mild OSA offers an opportunity to prevent cardiovascular deterioration, whereas delayed intervention may lead to progression to severe disease with significant health consequences. The presence of related comorbidities, especially cardiovascular conditions, should prompt clinicians to screen for OSA [11].

Two studies examined the interaction between OSA and obstructive respiratory diseases, primarily COPD [15,68]. Soriano et al. found that 35.9% of the participants met diagnostic criteria for OSA based on AHI, while 12.8% were diagnosed with COPD according to FEV1/FVC ratios [68]. A subsequent study, using data from the Obstructive Lung Disease and OSA cohort, analyzed mortality associated with these conditions individually and in combination [15]. Mortality for OSA alone was slightly lower than for COPD (60.4% vs. 63.0%) [15]. However, outcomes worsened considerably when asthma coexisted with either COPD or OSA, with mortality rates of 53.2% for COPD–OSA, 62.1% for asthma–COPD, 63.5% for asthma–OSA, and 67.8% for asthma–COPD–OSA [15].

Neurological disorders may also influence the progression of OSA. A recent meta-analysis reported that nearly half of the 1448 participants with neurological conditions also had OSA, with the severity of motor symptoms being a key linking factor [82]. In addition to Parkinson’s disease, a causal relationship has been identified between OSA and amyotrophic lateral sclerosis (ALS) [83]. In ALS, diaphragmatic dysfunction renders OSA more problematic than central sleep apnea, leading to worsened nocturnal hypoventilation and excessive daytime sleepiness [84,85]. ALS is inherently associated with sleep fragmentation, which further aggravates daytime sleepiness [84]. Despite these associations, the natural history of OSA in neurological disorders remains understudied, leaving critical gaps in understanding how early recognition and targeted management could modify outcomes.

Interestingly, excessive daytime sleepiness does not necessarily follow a linear course throughout OSA progression [53]. In a 5-year longitudinal study, participants were divided according to symptoms into four groups: minimally symptomatic, disturbed sleep, moderately sleepy, and excessively sleepy [53]. Over time, older participants tended to transition from excessive to moderate sleepiness, while women were more likely to shift from moderate sleepiness to minimal symptoms [53]. Lyyra et al. found that objective polygraphy indices of OSA severity were comparable across age groups (<70, 70–80, and >80 years), but older adults reported more subjective symptoms, including fatigue and mental distress [86]. These findings suggest that while objective disease severity may remain stable with age, subjective burden and HRQoL decline progressively when OSA remains untreated. Age-related physiological changes, including reduced upper airway muscle tone, increased pharyngeal fat deposition, enhanced airway collapsibility, and sleep fragmentation, further exacerbate OSA severity in older adults [87].

#### 3.3.4. Treatment-Modified History of Adults with OSA

The introduction of PAP therapy in 1981 marked a turning point not only in the management of OSA [21] but also in how its natural history can be observed and interpreted, by profoundly modifying disease trajectories and long-term outcomes. This transformative impact was confirmed by Ioachimescu et al. [15], who demonstrated that untreated OSA was associated with a significantly higher risk of death compared to patients receiving PAP therapy. Specifically, the unadjusted hazard ratio for mortality was 2.86 (95% CI: 2.46–3.33, *p* < 0.001), and after adjustment for potential confounders, the risk remained elevated (1.34; 95% CI: 1.05–1.71, *p* < 0.001) [15,66]. These findings underscore the importance of early diagnosis and timely initiation of PAP therapy in modifying disease trajectory and improving survival outcomes.

Although targeted pharmacotherapy and anti-obesity medications represent emerging therapeutic avenues, CPAP therapy remains the cornerstone of OSA management [16,88]. CPAP therapy significantly reduces AHI and downstream morbidity; however, its effects are primarily functional and exposure-dependent, whereas most non-CPAP interventions show less consistent or durable effects on long-term severity indices. CPAP therapy significantly reduces AHI, whereas most non-CPAP interventions show less consistent or even adverse long-term effects on AHI [89]. Beyond symptom control, CPAP use has been shown to improve all-cause and cardiovascular mortality. A recent large-scale meta-analysis including 1175,615 individuals confirmed that CPAP therapy substantially prolongs life expectancy in patients with OSA [7].

Mandibular advancement devices (MADs) offer an alternative treatment option and effectively reduce AHI, though typically to a lesser extent than CPAP. A 10-year follow-up study showed that MAD therapy reduced AHI from 31.7 ± 20.6 to 9.9 ± 10.3 events/hour, while CPAP achieved a more pronounced reduction from 49.2 ± 26.1 to 3.4 ± 5.4 events/hour [90]. However, MAD therapy can be associated with adverse effects, including occlusal changes and temporomandibular joint discomfort, particularly within the first two years of use [91]. A meta-analysis and a subsequent 10-year longitudinal study further confirmed the potential for dental and skeletal alterations with long-term use [92,93].

Hypoglossal nerve stimulation (HNS), first introduced in 2001 as a treatment option for OSA, has shown promising results in recent years [94]. Evidence suggests that HNS may provide greater improvements in patient-reported outcomes, such as excessive daytime sleepiness, assessed using the ESS, compared to PAP therapy [95]. Importantly, these benefits persisted over time, with one-year outcomes comparable to those observed after three months of PAP therapy [95]. Despite these encouraging findings, data on HNS and other alternative therapies remain limited regarding their long-term effects on disease progression and natural history. Further longitudinal studies are needed to determine whether these interventions can sustainably modify OSA outcomes.

However, other modifications, such as weight reduction and lifestyle behaviors, also represent effective strategies for mitigating OSA severity, as they play a critical role in its onset, progression, and management. Among these, alcohol consumption, smoking, sedative use, and obesity are particularly influential [96].

Alcohol consumption is a well-established contributor to OSA severity. Early evidence from a 1982 case-control study demonstrated that nocturnal alcohol intake markedly exacerbated OSA symptoms [97]. More recent evidence has reinforced this association, revealing a significant correlation between alcohol consumption and AHI, particularly among women [98]. Several physiological mechanisms may explain this relationship. Alcohol decreases upper airway muscle tone, increases pharyngeal collapsibility, and impairs arousal responses to apnea, thereby worsening SDB. It may even convert benign snoring into clinically significant OSA in susceptible individuals [87]. Alcohol-related OSA may result primarily from oropharyngeal muscle hypotonia, while impaired arousal mechanisms appear to contribute to disease persistence [99]. From a clinical perspective, these findings support routine assessment of alcohol intake in patients evaluated for OSA. Patients should be counseled on the potential exacerbating effects of evening or nocturnal alcohol consumption, particularly those with moderate-to-severe disease or residual symptoms despite treatment. Reducing or avoiding alcohol intake before sleep may represent a simple, low-cost adjunctive strategy to mitigate OSA severity and improve treatment outcomes.

Cigarette smoking has also been linked to OSA through its inflammatory effects on the upper airway, particularly within the uvula. These changes are mediated by calcitonin gene-related peptide-expressing neurons, which promote local tissue oedema and airway narrowing [100]. Smoking status should be systematically evaluated in patients with suspected or confirmed OSA. Given the inflammatory contribution of smoking to upper airway narrowing and clinical burden, smoking cessation should be encouraged as part of a comprehensive OSA management plan. Although cessation alone may not fully reverse OSA, it may reduce symptom burden, improve upper airway health, and confer substantial cardiovascular and respiratory benefits.

The role of sedatives in OSA progression is complex. Some agents, such as eszopiclone and sodium oxybate, have been shown to reduce AHI, whereas others, including remifentanil, zolpidem, and triazolam, may exacerbate the condition, as highlighted in a Cochrane meta-analysis [101]. Thus, although certain sedatives may hold therapeutic potential, their use in patients with symptomatic OSA requires caution due to the risk of impaired arousal responses, worsened respiratory events, and increased accident risk [102]. Clinicians should carefully review sedative use in patients with OSA, balancing potential benefits against risks. Additionally, the dosage and chronicity of use should be evaluated when presented with complex sleep apnea cases and excessive daytime sleepiness.

Obesity is widely recognized as one of the most important modifiable risk factors for OSA [22]. Pinto et al. [66] found a significant correlation between obesity and OSA severity, with higher BMI strongly associated with severe OSA. This relationship is especially pronounced in individuals with type 2 diabetes mellitus (T2DM), where excess weight enhances both metabolic and respiratory dysfunction [103]. Nevertheless, OSA can also occur in non-obese individuals, where it remains associated with impaired glucose metabolism and an increased risk of diabetes and cardiovascular disease [12]. This highlights that while obesity is a major contributor, OSA pathophysiology extends beyond body weight alone.

**Figure 3:**
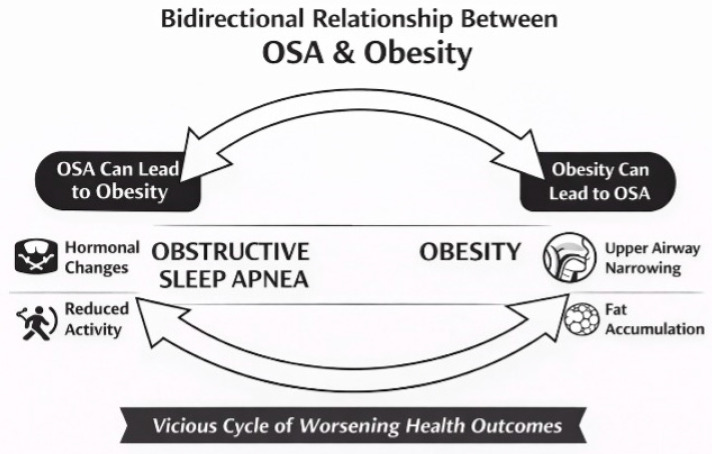
Bidirectional relationship between OSA & obesity.

Interestingly, in obese patients with OSA, glucose dysregulation may not always align with traditional classifications, such as impaired fasting glucose or glucose tolerance [12]. A meta-analysis involving over 338,000 individuals demonstrated a linear relationship between OSA severity and T2DM risk [13]. These findings support a bidirectional relationship between OSA and T2DM: intermittent hypoxemia and sleep fragmentation in OSA promote glucose dysregulation, while diabetic neuropathy impairs respiratory control and suppresses upper airway reflexes, further aggravating OSA [104] (Figure 3).

The interaction between CPAP therapy and body weight is complex. While weight loss consistently improves OSA severity, evidence supports that CPAP therapy may inadvertently contribute to weight gain [65]. A meta-analysis of 6954 individuals reported that CPAP use for more than four weeks significantly increased BMI as well as waist and neck circumference [28]. Notably, weight gain was more pronounced in patients using CPAP for less than five hours per night, whereas longer nightly adherence appeared to attenuate this effect [28]. Baseline cardiovascular disease and poor glycemic control were associated with greater BMI increases, suggesting that comorbidities influence CPAP-related weight changes. A recent study found that CPAP therapy significantly improved several parameters of metabolic syndrome, namely body weight, hepatic steatosis, lipid profile, adiponectin, and leptin, in patients with OSA. However, despite these improvements, most patients continued to meet diagnostic criteria for metabolic syndrome after six months of treatment, underscoring CPAP’s limitations in addressing metabolic risk without concurrent lifestyle modification [105].

Although some studies, such as that by Ou et al., failed to observe significant long-term weight changes, others reported modest weight gain [106]. For instance, Quan et al. found that CPAP users gained an average of 0.35 ± 5.01 kg, with each additional hour of nightly use associated with a 0.42 kg weight increase [107]. Paradoxically, patients who adhered to CPAP for more than four hours per night gained more weight than non-adherent users, while those using sham CPAP experienced modest weight loss (0.70 ± 4.03 kg), highlighting the complex metabolic effects of CPAP therapy [107].

**Table 3:** Integrated comparison of adult OSA natural history across population groups.

Population Group	Baseline Phenotype (Typical)	Factors Reported Alongside Higher Severity/Persistence or Adverse Trajectory *	Trajectory Patterns Reported in Longitudinal Evidence	Longitudinal Evidence Base (Study Type & Follow-Up)	Quantitative Findings Reported
Non-obese adults with OSA	OSA can occur in non-obese adults and may reflect anatomical/physiologic predisposition; metabolic risk may still be present despite normal BMI [12].	Aging and incremental weight gain; cardiometabolic comorbidity burden and metabolic dysfunction have been reported alongside higher risk/impact [12,13,104,105].	Adult OSA is often stable or slowly progressive with heterogeneity by phenotype and comorbidity context [65,67,68].	General adult cohorts and clinical follow-ups; ~5-year follow-up is common; samples and baseline severity vary widely [65,66,67,68].	Prevalence estimates in adult populations summarized as ~17% women and ~34% men [52]. Untreated OSA associated with higher mortality versus PAP-treated in cohort analyses (including OLDOSA: unadjusted HR 2.86; adjusted HR 1.34) [15,66]
Men (middle-aged)	Higher prevalence and typically higher AHI than women pre-menopause; more “classic” symptom patterns reported in screening/clinical contexts [52,56,57,58].	Weight gain/obesity and anatomical collapsibility; hypogonadism/low testosterone has been described as bidirectionally linked with OSA and adiposity [61].	Incidence increases with age until ~65, then plateaus; symptom burden can shift over time, not always mirroring objective indices [53].	Population-based and cohort studies, including symptom-subtype trajectory work over ~5 years [53].	Adult prevalence summarized as ~34% in men [52]. In symptom-subtype transitions over 5 years, older age increased the odds of shifting from “excessively sleepy” to “moderately sleepy” (per 5 years older: OR 1.52) [53].
Women (premenopausal)	Lower prevalence/severity than age-matched men; symptom presentation can be less classic in some cases [52,56,57,58].	Weight gain and sleep-medication/sedative exposures are discussed in the lifestyle literature; comorbid insomnia/depression may shape presentation and detection [64,101,102].	Often stable in general cohorts, with risk increasing as weight and hormonal milieu shift approaching menopause [59,62].	Cohorts including symptom-subtype transitions and epidemiologic observations across adult age ranges [53,59,62].	In 5-year symptom-subtype transitions, women had higher odds of moving from “moderately sleepy” to “minimally symptomatic” (OR 1.97) [53].
Postmenopausal women	Incidence and severity rise after menopause, approaching male levels; symptoms frequently include insomnia/fatigue/mood disturbance rather than classic snoring/witnessed apneas [59,62,63,64].	Decline in estrogen/progesterone and increased visceral adiposity (often independent of overall BMI) are repeatedly cited in mechanistic/epidemiologic discussions [59,62].	Higher likelihood of underdiagnosis when presentation is atypical; progression risk is commonly framed in relation to adiposity and hormonal transition [59,62,63,64].	Epidemiologic and clinical cohorts around menopausal transition; limited long-term, untreated follow-up focused solely on this group [59,62,63,64].	Reviews summarize that in postmenopausal women, nocturia may be under-reported but may be a hallmark OSA symptom in this population [64].
Older adults (≥65 years)	Objective severity indices may be comparable across older age strata, but symptom burden (fatigue/mental distress) and HRQoL impact can be greater [87].	Age-related reductions in upper airway muscle tone, increased pharyngeal fat deposition, and sleep fragmentation; multimorbidity context [87].	Physiologic severity may be relatively stable across age strata while subjective burden/HRQoL may worsen when untreated [86,87].	Cross-sectional comparisons (<70, 70–80, >80) plus limited longitudinal follow-up embedded within broader cohorts [86].	Across age strata, objective polygraphy indices reported as comparable, while older adults reported higher subjective symptoms (fatigue/mental distress) [86].
Obese individuals	Moderate–severe OSA is common; metabolic syndrome/T2DM and cardiometabolic risk frequently co-occur [66,103].	Visceral adiposity and metabolic dysfunction; OSA–T2DM bidirectional mechanisms (intermittent hypoxemia/sleep fragmentation; dysglycemia/neuropathy) [13,103,104].	Highest risk of worsening with weight gain; strongest evidence for improvement with sustained weight loss interventions [108,109,110].	Longitudinal weight-change studies; bariatric surgery and anti-obesity pharmacotherapy cohorts (months to years) [108,109,110,111,112,113].	20% weight reduction resulting in ~57% decrease in AHI (plateau beyond 20%) [108]. ≥5% weight loss within 1 year resulting in ~80% reduction in progression over 5 years [109]. Bariatric surgery: ~65% remission at 1 year [110]. GLP-1RA therapy: mean AHI decrease ~9.48 events/h [111]; additional anti-obesity pharmacotherapy evidence summarized (including tirzepatide) [112] and SGLT-2 inhibitors [113].

* Factors are reported alongside severity/persistence/progression in the included evidence; the table maps patterns other than causality, and longitudinal inference depends on the underlying study designs summarized in the Results.

Weight reduction is among the most effective strategies for mitigating OSA severity. A 2024 meta-analysis concluded that a 20% weight reduction led to a 57% decrease in AHI, with no additional benefit beyond this threshold, suggesting a plateau effect [108]. Similarly, a longitudinal study reported that even a 5% weight loss within one year was associated with an 80% reduction in OSA progression over a five-year period [109]. Collectively, these findings emphasize the importance of early and sustained weight management in preventing OSA progression and improving HRQoL [108,109,110].

Bariatric surgery has proven particularly effective in addressing obesity-related OSA. A recent cohort study by Al Oweidat et al. showed significant reductions in both AHI and respiratory disturbance index (RDI) one year after surgery, with 65% of patients achieving OSA remission. These findings illustrate bariatric surgery’s potential to substantially alter disease trajectory in obese patients [110].

Recent advances in anti-obesity pharmacotherapy have also shown promising results in the management of patients with OSA. Glucagon-like peptide 1-receptor agonists (GLP-1RAs), originally developed for diabetes treatment, have been associated with significant reductions in AHI. Li et al. reported a mean AHI decrease of 9.48 events/hour in patients receiving GLP-1 RAs, accompanied by improvements in body weight and blood pressure [111]. Tirzepatide, a dual GLP-1 RA/gastric inhibitory peptide agonist, has similarly demonstrated marked reductions in AHI among patients with OSA and obesity [112]. Comparable results have been reported with sodium-glucose cotransporter-2 inhibitors, suggesting a broader pharmacological potential in obesity-related OSA management [113]. These agents represent a promising adjunct or alternative to CPAP, particularly in obese patients, although large-scale randomized controlled trials are still needed to confirm efficacy and guide future clinical recommendations [113].

## 4. Discussion

The present work provides a comprehensive overview of current knowledge on the natural history of OSA. This narrative review illustrates how developmental stage, anatomy, lifestyle, and comorbidities shape disease onset, progression, and outcomes. Most studies suggest that early recognition and intervention can decisively alter the disease trajectory, improving HRQoL and reducing long-term systemic risks [7,8].

In children, OSA most commonly presents between ages 2 and 9, often due to adenotonsillar hypertrophy. However, risk factors such as obesity, rapid weight gain, prematurity, and African American ethnicity increase the likelihood of persistence or recurrence [18,21,22]. Longitudinal data indicate that although many children remit spontaneously, adolescence often represents a turning point, with worsening symptoms particularly in males, those with obesity, or those with tonsillar hypertrophy [23,26,27]. Notably, specific phenotypes, such as REM-predominant OSA, have been associated with adverse cardiovascular outcomes, including hypertension, emphasizing the importance of early identification [27,29]. Sex differences are minimal in childhood, but by adolescence, males are more likely to progress to severe disease, while females are more likely to experience remission [26,30,31]. These findings support phenotype- and sex-specific approaches to risk stratification.

Management of pediatric OSA is complicated by the high rate of spontaneous remission. For mild disease without impaired HRQoL, a “watchful waiting” approach may be appropriate [31,32]. However, for moderate-to-severe cases, adenotonsillectomy remains the first-line treatment, consistently improving sleep parameters and HRQoL [34,37,45]. Persistent OSA following surgery is often associated with obesity or craniofacial abnormalities and may require CPAP, pharmacological therapy, or more invasive surgical or orthodontic interventions [40,41,43,44]. Ensuring adherence to early intervention and structured follow-up is challenging. Long-term studies highlight that untreated pediatric OSA is associated with adult cardiovascular morbidity, poorer academic performance, and hypertension [48,49]. Therefore, early intervention in childhood has the potential to reshape long-term outcomes.

In adults, OSA is highly prevalent, affecting up to one-third of middle-aged men and nearly one-fifth of women [52]. Symptoms such as loud snoring, sleep fragmentation, and excessive daytime sleepiness are frequently unrecognized, delaying diagnosis until the disease has progressed [56,57,58]. Screening tools such as the STOP-BANG questionnaire and the Epworth Sleepiness Scale are valuable for risk stratification and can assist in early recognition [57,58]. Untreated OSA substantially reduces quality of life [70,71], increases workplace disability and accident risk [69,70,71,72,73], and contributes to the progression of cardiovascular and metabolic disease [76,77,78,79,114]. Importantly, comorbidities such as hypertension or diabetes can both accelerate and be exacerbated by OSA, creating a vicious cycle of disease progression [11,80,81]. Growing evidence suggests that combining questionnaires with objective or semi-objective data (snoring event severity, anthropometric measures, and data from additional sensors) can enhance predictive accuracy [115].

Further refinement of population risk stratification may come from understanding the interplay between sex and age in the natural history of OSA. Men remain at higher risk throughout life, but the incidence in women rises sharply after menopause, driven by hormonal decline and increased visceral adiposity [59,62]. Postmenopausal women frequently present with atypical symptoms, namely insomnia, fatigue, and mood disturbances, rather than the classic presentation of snoring and witnessed apneas, leading to underdiagnosis [63,64]. In men, hypogonadism and low testosterone levels can both exacerbate and worsen OSA, further complicating management [61]. Aging also influences disease progression: while severity indices may remain stable, older adults often experience increased sleep fragmentation, psychological distress, and reduced HRQoL [53,55,86].

Across the literature, obesity consistently emerges as the most important modifiable risk factor. Higher BMI strongly predicts disease severity, while weight loss reliably improves OSA indices [66,103,104,108,109,116]. Even modest reductions in body weight can reduce progression risk by up to 80% [97]. Bariatric surgery achieves remission in many obese patients [110], while newer pharmacological agents, such as GLP-1 receptor agonists, also show promising reductions in AHI [15,88]. Interestingly, while CPAP is highly effective for symptom control and mortality reduction, it may contribute to modest weight gain, particularly in poorly adherent patients [28,107]. These findings reinforce the necessity of coupling CPAP therapy with active weight management for sustained benefits.

CPAP remains the cornerstone of OSA management, with robust evidence demonstrating reductions in cardiovascular and all-cause mortality [14,81,88]. It should be emphasized, however, that the widespread availability of modern treatments has ironically made genuine natural history research more difficult from an academic perspective. Alternatives such as MADs [90,91,92,93] and HNS [94,95] provide effective options for selected patients, although their long-term impact on disease progression remains less certain. Compared with untreated OSA, long-term MAD use is associated with lower symptom progression and fewer intermediate adverse outcomes. However, MADs do not completely mitigate underlying contributors, such as aging, weight gain, and craniofacial anatomy. Recent systematic reviews suggest that these interventions stabilize or slow disease progression in responders rather than achieve permanent reversal [117]. To date, only CPAP has consistently demonstrated modification of long-term outcomes, reinforcing the importance of early initiation and sustained adherence.

From a clinical and pathophysiological perspective, the impact of therapeutic interventions on the natural history of OSA varies substantially and depends on both the mechanism targeted and the durability of treatment adherence. CPAP remains the most effective therapy for immediate alleviation of respiratory events and reduction in cardiovascular risk. Its effect, however, is primarily functional and contingent on sustained use, with disease severity typically re-emerging upon discontinuation. In contrast, interventions that address upstream drivers of OSA and particularly excess body weight and metabolic dysfunction appear to exert more durable, disease-modifying effects. Their benefits, however, are closely linked to long-term adherence to weight maintenance. Emerging modalities, such as pharmacologic anti-obesity therapies and hypoglossal nerve stimulation, expand the therapeutic landscape but are effective only in carefully selected phenotypes. Therefore, they require further longitudinal documentation of their efficacy. These evidence-based observations demonstrate the importance of individualized treatment selection based on disease severity, phenotype, comorbidities, and patient preferences. Equally critical is structured longitudinal follow-up, as OSA is a dynamic disorder influenced by aging, weight changes, hormonal transitions, and evolving comorbidities. Regular and structured reassessment allows timely treatment adjustment, identification of relapse or progression, and integration of adjunctive interventions, thereby optimizing long-term outcomes beyond short-term symptom control.

Significant gaps remain in our understanding of the natural history of untreated adult OSA. The longitudinal progression of subjective symptom burden relative to objective polysomnographic measures is not fully characterized, particularly across different age groups and phenotypes [53,86]. The influence of specific comorbidities, especially non-cardiovascular conditions like neurological disorders, on the rate of OSA progression is understudied, and existing research is often limited by inadequate adjustment for key confounders such as age and baseline disease severity [65,82,84]. Furthermore, the long-term trajectory of OSA in specific populations, including non-obese individuals and postmenopausal women presenting with atypical symptoms, requires further clarification to guide targeted screening and understand prognosis [12,63]. Finally, the precise mechanisms driving the observed disconnect between stabilized objective indices and worsening patient-reported outcomes in aging populations remain to be elucidated [86,87].

Besides the classic observational or interventional longitudinal cohorts, artificial intelligence (AI) prediction models could help stratify the risk and guide the screening and treatment of specific populations. Recent systematic reviews and original studies have shown that machine learning (ML) and deep learning (DL) algorithms can predict the presence and severity of OSA with promising performance metrics [118]. In addition to binary detection or risk stratification, AI models have been used to predict CPAP adherence and therapeutic outcomes using ML classifiers [119]. Moreover, integration of panels combining inflammatory, oxidative, metabolic, and emerging molecular markers with AI-driven models and longitudinal clinical data may enable a more personalized and dynamic approach to OSA management. Therefore, AI has a growing but still emerging role in understanding and shaping the natural history of OSA. However, at present, it should be viewed as a complementary tool that may support personalized monitoring and adaptive management, rather than being a standalone determinant of OSA natural history.

## 5. Conclusions

The natural history of OSA remains incompletely understood, largely due to the scarcity of long-term longitudinal data. At present, diagnosis often occurs in the later stages of the disease, after HRQoL and work performance have already been compromised. This diagnostic delay allows for progression to severe OSA, in which HRQoL is markedly reduced, and systemic health is further impaired by comorbidities. Early identification through targeted symptom screening and the use of validated questionnaires may enable the detection of OSA before the onset of severe complications and comorbidities. Timely intervention, particularly in mild OSA, has the potential to alter the disease course and preserve HRQoL. The therapeutic landscape is also expanding rapidly, allowing for personalized and effective management strategies that can modify disease progression and reduce comorbidity risk. Despite these advances, CPAP remains the only intervention consistently proven to improve long-term outcomes. Weight management, along with bariatric or pharmacological therapies, offers substantial benefits, especially in obesity-related OSA. Prioritizing early identification and intervention across the lifespan represents the most effective strategy to mitigate OSA’s long-term impact and preserve overall quality of life. Prospective studies integrating AI- or biomarker-driven risk prediction with long-term clinical follow-up are needed to determine whether the actions of clinicians can meaningfully modify disease trajectories and long-term outcomes.

## Figures and Tables

**Table 1:** Summary of included charted longitudinal studies.

Study (Author, Year)	Country/Setting	Population	OSA Definition & Baseline Severity	Study Design & Follow-Up	Natural History Outcomes (Direction)	Key Predictors Reported	Main Findings	Limitations
Ali et al. 1994	UK; community cohort	Children aged 4–7 years; N = 782 baseline, 507 follow-up	Habitual snoring by parent report; severity not PSG-defined	Longitudinal observational; 2 years	Snoring persistence and resolution; both remission and new onset observed	Age-related maturation	Over half of habitual snorers no longer snored at follow-up; overall prevalence remained stable due to incident cases	Symptom-based; no PSG
Spilsbury et al. 2015	USA; community cohort	Children aged 8–11 years at baseline, 16–19 years at follow-up; N = 490	PSG-defined OSA (OAHI ≥ 5 or OAI ≥ 1); mixed severity	Prospective cohort; mean 8.2 years	OSA remission and incidence	Sex, body mass index, adenotonsillectomy	Most childhood OSA resolved by adolescence; approximately 4% developed new adolescent OSA	Different sleep assessment methods across timepoints
Bixler et al. 2016	USA; population cohort	Prepubertal children followed into adolescence; N = 700 baseline, 421 follow-up	PSG-defined SDB (AHI ≥ 2 and ≥5); mixed severity	Longitudinal cohort; approximately 8 years	Remission of childhood SDB and adolescent incidence	Sex, obesity, age	Complete remission of childhood AHI ≥ 5; substantial adolescent incidence of SDB	Attrition over follow-up
Li et al. 2010	Hong Kong; community cohort	Children aged 6–13 years; N = 56 baseline, 45 follow-up	PSG-defined mild OSA (OAHI 1–5)	Prospective cohort; 2 years	Progression and remission of mild OSA	Central obesity, tonsil size, sex	Nearly one-third showed worsening OAHI; progression associated with obesity and tonsillar hypertrophy	Small sample
Chan et al. 2019	Hong Kong; community cohort	Children aged 6–13 years followed into young adulthood (16–25 years); N = 243	PSG-defined OSA (child OAHI ≥ 1; adult OAHI ≥ 5); mild to severe	Prospective cohort; mean 10.4 years	Remission, persistence, and incident adult OSA	Sex, body mass index	Thirty percent achieved full remission; twenty-two percent developed adult OSA	Attrition
Chan et al. 2020	Hong Kong; cohort	Same cohort as above; N = 243	PSG-defined childhood OSA; mixed severity	Prospective cohort; approximately 10 years	Long-term cardiovascular consequences	Childhood OSA severity	Childhood OSA independently predicted higher adult nocturnal blood pressure	Cardiovascular outcomes only
Chan et al. 2021	Hong Kong; cohort	Children aged 6–13 years followed into adulthood; N = 619 baseline, 234 follow-up	PSG-defined REM-predominant OSA; mixed severity	Prospective cohort; approximately 10 years	Stability of REM-related OSA phenotype	Body mass index, REM predominance	REM-related OSA was common and largely stable; associated with adverse blood pressure profiles	Attrition
Amin et al. 2008	USA; tertiary pediatric center	Children aged 7–13 years post-adenotonsillectomy; N = 40	PSG-defined OSA (AHI > 1); mixed severity	Prospective post-surgical cohort; 1 year	Recurrence of OSA after initial improvement	Weight gain, obesity	Half of children experienced recurrent OSA within one year	Post-treatment course
Nosetti et al. 2022	Italy; sleep center	Children diagnosed at mean age ~5 years; N = 100 at adult follow-up	PSG-confirmed severe childhood OSA; adult outcomes by questionnaire	Case–control; approximately 20 years	Persistence of symptoms and long-term sequelae	Childhood AHI, body mass index	Severe childhood OSA associated with higher adult BMI, snoring, and lower educational attainment	No adult PSG
Pendlebury et al. 1997	UK/France; sleep clinic	Adults; N = 55	PSG-defined mild to moderate OSA	Retrospective cohort; mean 17 months	Progression of AHI over time	None identified	More than half progressed to require treatment	Adult clinic cohort
Fisher et al. 2002	Israel; sleep clinic	Adults; N = 40	PSG-defined OSA (RDI-based); mixed severity	Observational cohort; approximately 5 years	Change in the respiratory disturbance index	Body mass index	Respiratory indices remained largely stable; cardiovascular disease developed in untreated patients	Small sample
Morris et al. 2024	USA; SHHS	Adults aged 40 years and older; N = 2619	PSG-defined OSA (AHI ≥ 5); mixed severity	Longitudinal cohort; mean 5.2 years	Symptom subtype transitions	Body mass index, sex	Nearly half transitioned between symptom subtypes over time	Adult population
Sforza et al. 1994	Italy; sleep clinic	Adults refusing treatment; N = 32 with instrumental follow-up	PSG-defined OSA; mixed severity	Prospective longitudinal; mean 5.7 years	Stability of AHI and hypoxaemia	None identified	Apnea frequency and hypoxaemia largely unchanged; event duration increased	Small untreated cohort
Soriano et al. 2010 (PULSAIB)	Spain; population cohort	Adults aged 30–80 years; N = 305	Home respiratory polygraphy (AHI > 10); mixed severity	Cross-sectional baseline of cohort	Prevalence and framework for future natural history	Age, sex, obesity	High prevalence of OSA; feasibility of population cohort demonstrated	-

**Table 2:** Summary of factors and outcomes in pediatric and adolescent OSA.

Category	Findings/Characteristics
Anatomic Factors	Adenotonsillar hypertrophy (most common etiology); craniofacial abnormalities (micrognathia, retrognathia); neuromuscular dysfunction; airway resistance abnormalities.
Demographic Factors	Predominant age range: 2–8 years (can extend to adolescence). Male sex predicts persistence and severity; female sex often associated with remission. African American ethnicity and lower socioeconomic status increase risk.
Physiological/Developmental	REM-predominant OSA common; linked to elevated blood pressure and cardiovascular risk in adolescence. Transition to adolescence often leads to worsening symptoms, particularly in males and obese children.
Lifestyle/Behavioral	Childhood obesity and rapid weight gain strongly associated with the development and recurrence of OSA post-adenotonsillectomy; sedentary lifestyle and poor diet are key modifiable risks.
Comorbidities	Hypertension (especially in REM-OSA); obesity; neurobehavioral and cognitive dysfunction; learning and attention deficits; emotional or internalizing behavior problems, particularly in females with moderate-to-severe OSA.
Genetic/Familial Factors	Familial aggregation reported; craniofacial anatomical traits may be inherited. 43% of first-degree relatives exhibit OSA-like symptoms despite few formal diagnoses.

## Data Availability

No new data were created.

## Supplementary Materials

The following supporting information can be downloaded at: https://www.mdpi.com/article/10.3390/healthcare14030325/s1, File S1: Search strategy and eligibility framework.

## Abbreviations

The following abbreviations are used in this manuscript:

AHI	apnea–hypopnea index
ALS	amyotrophic lateral sclerosis
BMI	body mass index
CHAT	Childhood Adenotonsillectomy Trial
CI	confidence interval
COPD	chronic obstructive pulmonary disease
CPAP	continuous positive airway pressure
ESS	Epworth Sleepiness Scale
FEV_1_	forced expiratory volume in 1 s
FVC	forced vital capacity
GLP 1RA(s)	glucagon-like peptide-1receptor agonist(s)
HNS	hypoglossal nerve stimulation
HRQoL	health-related quality of life
MADs	mandibular advancement devices
MeSH	medical subject headings
OAHI	obstructive apnea-hypopnea index
OSA	obstructive sleep apnea
OSA-18	OSA-18 questionnaire
PAP	positive airway pressure
RDI	respiratory disturbance index
REM	rapid eye movement
REM-OSA	rapid eye movement-predominant obstructive sleep apnea
SDB	sleep-disordered breathing
T2DM	type 2 diabetes mellitus
USA	United States of America
WWSC	watchful waiting and supportive care
AI	artificial intelligence
ML	machine learning
DL	deep learning
