# Supplementary material for: The Natural History of Obstructive Sleep Apnea: A Scoping Review
Source: Healthcare (Basel). 2026 Jan 27;14(3):325. doi: 10.3390/healthcare14030325 (PMC12897078; doi:10.3390/healthcare14030325)
Supplement: Supplementary file 1 [file healthcare-14-00325-s001.zip › healthcare-4018205-supplementary.pdf]

Supplementary Material S1. Search strategy and eligibility framework

S1. Search scope and rationale

Given the breadth of the topic and the original narrative scope of the work, **no date limits or study-design limits** were applied at the search stage. Searches were conducted from **database inception to September 2025** and were designed to capture evidence relevant to the **life-course evolution of obstructive sleep apnea (OSA)** across **pediatric and adult populations**.

Search strategies combined controlled vocabulary (where available) with free-text terms to maximize sensitivity for longitudinal, trajectory-focused, and modifier-informed evidence. Study-design restrictions were deliberately avoided to allow capture of heterogeneous longitudinal cohorts, extended follow-up analyses, and natural history–relevant reports published across different eras of OSA research.

S2. Eligibility criteria

Inclusion criteria

Records were eligible for inclusion if they met all of the following criteria:

- Published in **English**
- **Peer-reviewed** journal articles
- Included **pediatric and/or adult** populations
- Contributed evidence relevant to the **life-course evolution of OSA**, informing at least one of the mapped domains below

Mapped eligibility domains

Studies were eligible if they informed one or more of the following domains:

1. **Trajectory evidence**
   Evidence addressing OSA onset, persistence, remission, progression, recurrence, or long-term sequelae plausibly linked to earlier OSA exposure.
2. **Determinants or modifiers of course**
   Evidence addressing factors that plausibly shape observed OSA trajectories or long-term outcomes, including (but not limited to):
   - Phenotypic features (e.g., REM-related OSA patterns)
   - Risk factors (e.g., adiposity measures, tonsillar or craniofacial factors)
   - Demographic modifiers (e.g., sex differences, menopausal transition)
   - Comorbidity interactions
   - Treatment-era considerations relevant to observed trajectories or outcomes

Exclusion criteria

- **Conference abstracts**, **editorials**, and **narrative opinion pieces** were excluded.
- **Narrative and systematic reviews** were not included as *primary evidence*, but were retained where relevant for **citation-chaining** and background framing.

S3. Citation-chaining

To enhance capture of relevant longitudinal cohorts and trajectory-focused analyses, **reference lists of key included studies and closely related reviews** were manually screened. Records identified through citation-chaining were subject to the same eligibility criteria as database-retrieved records.

S4. Database-specific search strategies

Conceptual structure

Search strategies across all databases were built around two core concept blocks:

- **Concept A (Condition):** obstructive sleep apnea / sleep-disordered breathing
- **Concept B (Life-course evolution):** natural history, longitudinal course, trajectory-related outcomes (e.g., persistence, remission), and determinants/modifiers of course (e.g., phenotypes, adiposity, demographic modifiers, comorbidity interactions, treatment era)

Concept B intentionally combined **trajectory terms** and **modifier terms**, reflecting the heterogeneity of terminology used to describe life-course evidence in OSA research.

S4.1 PubMed / MEDLINE

Database/platform: PubMed (NCBI)
Coverage: MEDLINE and PubMed-not-MEDLINE records
Date range: Database inception to September 2025
Limits applied: English language; no date or study-design limits

Search strategy:

```
(
  "Sleep Apnea, Obstructive"[Mesh]
  OR "obstructive sleep apnea"[tiab]
  OR "obstructive sleep apnoea"[tiab]
  OR OSA[tiab]
  OR OSAS[tiab]
  OR "sleep-disordered breathing"[tiab]
```

```
  OR "sleep disordered breathing"[tiab]
)
AND
(
  "natural history"[tiab]
  OR "disease course"[tiab]
  OR trajectory[tiab] OR trajectories[tiab]
  OR longitudinal[tiab]
  OR "follow-up"[tiab] OR "follow up"[tiab]
  OR cohort[tiab]
  OR incidence[tiab]
  OR onset[tiab]
  OR persistence[tiab] OR persistent[tiab]
  OR remission[tiab]
  OR progression[tiab]
  OR recurrence[tiab]
  OR sequelae[tiab]
  OR "Longitudinal Studies"[Mesh]
  OR "Cohort Studies"[Mesh]
  OR "Follow-Up Studies"[Mesh]
  OR "Incidence"[Mesh]
  OR "Time Factors"[Mesh]
  OR phenotype*[tiab]
  OR "REM-related"[tiab] OR "REM related"[tiab]
  OR adiposity[tiab] OR obesity[tiab] OR BMI[tiab]
  OR tonsil*[tiab] OR adenotonsil*[tiab]
  OR menopause[tiab] OR menopausal[tiab]
  OR comorbid*[tiab]
  OR treatment[tiab] OR CPAP[tiab] OR adenotonsillectomy[tiab]
)
```

S4.2 Scopus (Elsevier)

Database/platform: Scopus
Date range: Database inception to September 2025
Limits applied: English language; no date or study-design limits
Document filters: Journal articles; editorials, notes, letters, and conference materials excluded where feasible

Search strategy (TITLE-ABS-KEY):

```
TITLE-ABS-KEY(
  "obstructive sleep apnea" OR "obstructive sleep apnoea" OR OSA OR OSAS
  OR "sleep disordered breathing" OR "sleep-disordered breathing"
)
AND
TITLE-ABS-KEY(
  "natural history" OR "disease course"
  OR trajectory OR trajectories
  OR longitudinal OR "follow up" OR "follow-up" OR cohort
  OR incidence OR onset
  OR persistence OR persistent OR remission OR progression OR recurrence
  OR sequelae
  OR phenotype* OR "REM-related" OR "REM related"
  OR adiposity OR obesity OR BMI
  OR tonsil* OR adenotonsil*
  OR menopause OR menopausal
```

```
  OR comorbid*
  OR treatment OR CPAP OR adenotonsillectomy
)
```

S4.3 Google Scholar

Platform: Google Scholar
Purpose: Supplementary retrieval and citation-chaining
Sort order: Relevance
Screening cap: First *n* records (e.g., first 500), as specified in the PRISMA flow diagram
Settings: Patents and citations excluded

Search strategy:

```
("obstructive sleep apnea" OR OSA OR OSAS OR "sleep disordered breathing")
("natural history" OR trajectory OR trajectories OR longitudinal OR cohort
OR "follow up"
 OR incidence OR onset OR persistence OR remission OR progression OR
recurrence OR sequelae
 OR phenotype OR "REM-related" OR adiposity OR obesity OR BMI OR tonsil OR
adenotonsillectomy
 OR menopause OR comorbidity OR CPAP)
```

Records retrieved from Google Scholar were screened using the same eligibility criteria as database-retrieved records. Reviews were retained for citation-chaining but excluded as primary evidence.
